# Effect of Furfurylation on Bamboo-Scrimber Composites

**DOI:** 10.3390/ma16072931

**Published:** 2023-04-06

**Authors:** Wanju Li, Guijun Xie, Hongxia Ma, Xingwei Li

**Affiliations:** Guangdong Provincial Key Laboratory of Silviculture, Protection and Utilization, Guangdong Academy of Forestry, Guangzhou 510520, China

**Keywords:** physical properties, mechanical properties, durability, microstructure, thermostability

## Abstract

Bamboo is a material with excellent development prospects. It is increasingly used in furniture, decoration, building, and bridge construction. In this study, Furfurylated bamboo bundles and phenol-formaldehyde resin were used to make bamboo-scrimber composites (BSCs) via molding-recombination and hot-pressing processes. The effects of the impregnation mode, furfuryl-alcohol concentration, and curing temperature on the various physical–mechanical properties and durability of the composites were evaluated. Scanning-electron microscopy (SEM) was used to observe the microstructural differences. Fourier-transform infrared spectroscopy (FTIR) and X-ray photoelectron spectroscopy (XPS) were employed to investigate changes in the chemical constituents. The heat resistance was also investigated using thermogravimetric analysis. The results showed that the density of the furfurylated BSC increased by up to 22% compared with that of the BSC-C with the same paving mode. The furfurylated BSCs had lower moisture contents: the average moisture content of the furfurylated BSCs was 25~50% lower than that of the BSC-C. In addition, the furfurylated BSCs showed better dimensional stability and durability, since the decay-resistance grade of the BSCs was raised from decay resistance (class II) to strong decay resistance (class I). In terms of the mechanical properties, the furfurylation had a slight negative effect on the mechanical strength of the BSCs, and the modulus of rupture (MOR) and horizontal shear strength (HSS) of the BSCs were increased to a certain extent under most of the treatment conditions. In particular, the highest HSS for indoor use and MOR of the furfurylated BSCs increased by 21% and 9% compared with those of the untreated BSCs, respectively. The SEM results indicated that the FA resin effectively filled in the bamboo-cell cavities and vessels, and the modified bamboo-parenchyma cells were compressed more tightly and evenly. The FTIR and XPS spectroscopy showed that the hydroxyl group of carboxylic acid of the bamboo-cell-wall component reacted with that of the furan ring, and the cellulose and hemicellulose underwent acid hydrolysis to a certain extent after the furfurylation. Overall, the present study highlights the potential of furfurylation as a modification method to enhance BSC products. Further research should focus on improving the ability of furfurylated BSCs to prevent the growth of *Botryodiplodia theobromae*. Additionally, the influence of furfuryl-alcohol resin on the bonding strengths of PF adhesives should be further clarified.

## 1. Introduction

Bamboo is an important, sustainable, and abundant plant resource that has a shorter rotation and higher strength than most trees [1]. However, the smaller diameters and hollowness of bamboo have limited its use in the creation of sheets for decoration or as a load-bearing material for structural applications in building and construction [2]. To overcome this limitation, bamboo stems can be processed into bamboo strips, bamboo bundles, bamboo fiber mats, bamboo shavings, or bamboo fibers, and reconstituted to fabricate bamboo composites, such as bamboo plywood, laminated bamboo lumber, bamboo-scrimber composites, bamboo particleboards, and bamboo fiberboards [3,4]. In particular, bamboo scrimber is a type of high-performance, bamboo-based, reconstituted material with controllable performance and adjustable specifications and design structure. It is fabricated with bamboo bundles and phenol formaldehyde resin subjected to a specific pressure and temperature. Additionally, bamboo scrimber has a higher utilization rate, of up to 92.54%, compared with laminated bamboo lumber due to the use of the bamboo bundles without removing the outer wall of the bamboo [5]. In the past decades, researchers have conducted a significant amount of research by optimizing the unit material, adhesive content, and hot-pressing process, which have significantly improved the physical and mechanical properties of bamboo-scrimber composites [6,7,8,9]. Bamboo-scrimber material has been utilized in many fields, such as floor decking, furniture, structural materials, and wind-turbine blades [10].

However, natural bamboo, which is rich in sugar, starch, and protein, has a highly porous structure, which makes it vulnerable to mildew, fungi, and insects, deformation, and discoloration under the influence of changes in ambient temperature, humidity, and light conditions [11,12]. It is difficult to avoid these problems, even when relatively large amounts of adhesives are used in bamboo-scrimber composites. Therefore, modification or protective treatment are crucial for the efficient utilization of bamboo-scrimber composites, especially for those used in outdoor environments. Various methods have been employed; for example, Möller and Mild [13] found that copper amine and metal-free preservatives were useful for bamboo to prevent the brown-, white-, and soft-rot fungi and discoloration by mold fungi. Similarly, ammoniacal copper quaternary, copper triazole, a combination of propiconazole and tebuconazole, chitosan copper complex, and a combination of iodopropynyl butylcarbamate, propiconazole, and tebuconazole have also been employed to improve the anti-mildew properties of bamboo scrimber [14]. However, the adhesive-dipping process often leads to part of the anti-mildew agents being lost, reducing the efficacy of the anti-mildew agents and, consequently, increasing the potential risk of environmental pollution. Wu et al. [15] studied mesoporous aluminosilicate’s synergy with a Cu–B–P anti-mildew agent to improve the mildew resistance of bamboo scrimber and suggested that the mesoporous Si–Al complex is tightly wrapped around the bamboo-fiber surface, which is of great benefit for improving its leaching ability and enhancing the efficiency of anti-mildew agents. Furthermore, directly adding the protective agent to the adhesive is another effective method to treat bamboo scrimber; Fu et al. [16] used particles including CaCO_3_, CaO, MgCO_3_, and MgO as flame retardants and added them to phenolic resin, improving the physical, mechanical, and flame-retardant properties of a bamboo–wood-hybrid scrimber. It is worth noting that both the preservatives used to pretreat bamboo bundles and the preservatives added to adhesives need to have a certain heat resistance because the hot-pressing process is required in the manufacturing process of bamboo scrimber. Therefore, the reagents available for bamboo-scrimber protection are limited. Additionally, high-temperature heat treatment is highly recommended because it can reduce the hygroscopicity of bamboo and enhance its mildew-proof and anti-decay fungi properties. The dimensional stability and durability of bamboo scrimber can be significantly improved by heat treatment. However, the mechanical properties of bamboo scrimber decrease significantly due to the thermal degradation of hemicellulose [17,18,19].

Considering wood/bamboo-modification methods, furfurylation technology significantly improves the dimensional stability and durability of wood/bamboo [20,21,22,23,24,25,26]. This process impregnates furfuryl alcohol (FA), which has a strong polarity and is a low-molecular organic chemical derived from corncobs or sugar-cane residues, into wood/bamboo cavities and even cell walls. It is then thermally polymerized under the action of an acid catalyst. Previous studies found that the FA modifier is also very useful in improving the properties of bamboo, which is a material with poor permeability. The weight-gain rate of bamboo strips can be 12% after soaking treatment and 32% after vacuum-pressure impregnation [24,26].

As the unit materials of bamboo scrimber, bamboo bundles are obtained by rolling bamboo strips. It is reasonable to pre-modify them using furfurylation; however, how furfurylation affects the physical–mechanical properties, durability, and chemical constituents of bamboo scrimber needs to be clarified. The purpose of this study was to explore the feasibility of furfurylation for improving the performance of bamboo scrimber. Both soaking and vacuum–pressure–vacuum (V–P–V) impregnation processes and different FA concentrations were evaluated to optimize the treatment conditions for the furfurylation of bamboo scrimber. The equilibrium moisture content (EMC), density, water absorption (WA), thickness swelling rate (TSR), width swelling rate (WSR), outdoor and indoor horizontal shear strength (HSS), modulus of rupture (MOR), modulus of elasticity (MOE), resistance to mold, and decay fungi were evaluated. Furthermore, scanning-electron microscopy (SEM), Fourier-transform infrared spectroscopy (FTIR), and X-ray photoelectron spectroscopy (XPS) were employed to investigate changes in the microstructure and chemical composition. The heat resistance was investigated using thermogravimetric analysis (TGA).

## 2. Materials and Methods

### 2.1. Materials

Bamboo bundles were purchased from bamboo-flooring factory in Zhejiang Province, China. Phenol-formaldehyde (PF) resin was purchased from Guangdong Dynea Chemical Industry Co., Ltd. (Zhaoqing, China). Other agents, including furfuryl alcohol, sodium borate, citric acid, and oxalic acid, were purchased from Sinopharm Chemical Reagent Co., Ltd. (Shanghai, China).

### 2.2. Furfurylation of Bamboo Bundles

The furfurylation of bamboo bundles was slightly changed on the basis of the previous furfurylation of bamboo strips by Li et al. [24] and Liu et al. [26]. Bamboo bundles were impregnated with 30% and 50% FA solutions using soaking and vacuum–pressure–vacuum impregnation processes, respectively. In the soaking process, bamboo bundles were soaked in 50% FA solution for 24 h and 48 h under atmospheric pressure. The vacuum–pressure–vacuum impregnation process consisted of 10 min vacuum impregnation, followed by 1.2 MPa pressure impregnation for 10 min and then vacuum impregnation for 10 min. The impregnated bamboo bundles were left to sit for 4 days and then wrapped in aluminum foil for the curing stage. At this stage, the FA in the bamboo bundles was polymerized at 105 °C or 115 °C for 5 h. After polymerization, the modified bamboo bundles were further dried for 16 h at 80 °C and, subsequently, air-dried until the moisture content was below 10%. Specific sample information is shown in Table 1.

### 2.3. Preparation of Bamboo-Scrimber Composites

Bamboo-scrimber composites were fabricated according to Yu et al. [7], with a slight modification. Before dipping, PF resin with 52% solid content was diluted with water to 17.5% solid content. Bamboo bundles were completely soaked in PF resin for 11 min, placed horizontally for 4 min, and then laid flat to dry until the moisture content was less than 10%. The PF-resin dosage of bamboo bundles was controlled to 14% of the oven-dried weight of the bamboo bundles. Bamboo bundles were laid in five parallel layers and hot-pressed. Finally, bamboo-scrimber composites with dimensions of 450 mm × 550 mm × 18 mm were obtained.

### 2.4. Physical–Mechanical Properties

#### 2.4.1. Dimensional Stability

After a week of storage indoors, bamboo-scrimber composites were sawn into different sizes required for testing (see Table 2 for details). All specimens tested for physical and mechanical properties were conditioned to constant weight in a balancing chamber at a condition temperature of 20 ± 2 ℃ and humidity of 65 ± 5%. The TSR and WSR were used to evaluate the dimensional stability of bamboo scrimber, and the test method was based on GB/T 30364-2013 [27]. The 28-h treatment for exterior-use-test procedure was applied; namely, the thickness and width of the sample (accuracy ± 0.1 mm) were recorded before the test. The sample was soaked in boiling water for 4 h, dried in an oven at 63 ± 3 ℃ for 20 h, and soaked in boiling water for 4 h. Finally, the thickness and width of the specimens were recorded. The TSR and WSR were determined using Equations (1) and (2), respectively:

(1) $$\mathrm{TSR}(\%)=\frac{t_{1}-t_{0}}{t_{0}}\times 100\%$$

(2) $$\mathrm{WSR}(\%)=\frac{b_{1}-b_{0}}{b_{0}}\times 100\%$$

where *t*_0_ and *b*_0_ are the thickness and width, respectively, of the samples before the 28-h treatment, and *t*_1_ and *b*_1_ are the thickness and width, respectively, of the samples after the 28-h treatment.

#### 2.4.2. Horizontal Shear Strength

Mechanical-testing machine (AGS-X plus-50 kN, Shimadzu, Tokyo, Japan) was applied to test the horizontal shear strength of bamboo scrimber, according to the method in GB/T 30364-2013 [27]. For outdoor use, the specimens were subjected to a 28-h treatment, as described in Section 2.4.1, before mechanical testing. The horizontal shear strength was calculated using the following equation:

(3) $$\tau =\frac{3F}{4bh}$$

where *τ* is the HSS (MPa), and *F*, *b*, and *h* are the maximum failure load, width, and thickness of the specimen, respectively.

#### 2.4.3. Bending Strength and Modulus of Bamboo-Scrimber Composites

The MOR and MOE of the bamboo-scrimber-composite specimens were determined in a direction parallel to the face grains, according to GB/T 17657-2013 [28]. The specimens were tested using a three-point bending model with a bearing span of 280 mm and a loading speed of 8 mm/min. The MOR and MOE were calculated using Equations (4) and (5), respectively:

(4) $$MOR=\frac{3P_{\max}L}{2bh^{2}}$$

(5) $$MOE=\frac{P_{\rho }L^{3}}{4\delta bh^{3}}$$

where *P*_max_ is the maximum failure load of the sample (N), *L* is 280 mm, *b* and *h* are the width and thickness of the samples (mm), respectively, *P_ρ_* is the load increase (N) determined from the straight-line section of the load–deflection curve, and *δ* is the mid-span deflection (mm) of the sample under *P_ρ_*.

### 2.5. Biological Durability

#### 2.5.1. Anti-Mildew Property

Mold resistance was evaluated following the method described in GB/T 18261-2013 [29]. *Aspergillus niger* V. Tiegh, *Penicillium citrinum* Thom, *Trichoderma viride* Pers. ex Fr, and *Botryodiplodia theobromae* Pat were tested. Bamboo-scrimber samples were placed in culture dishes and incubated in an incubator at 22–25 °C and 85% RH for 4 weeks. Finally, the samples’ infected areas were recorded, and the infection values were judged, as shown in Table 3. The mold-prevention efficacy was calculated as follows:

(6) $$E=(1-\frac{D_{1}}{D_{0}})\times 100\%$$

where *E* is the mold-prevention efficacy (%), and *D*_0_ and *D*_1_ are the infection values of the untreated and furfurylated BSCs, respectively, after infection.

#### 2.5.2. Anti-Fungal-Decay Properties

Fungal-decay resistance was evaluated according to GB/T 13942.1-2009 [30]. The white-rot fungus *Coriolus versicolor* and the brown-rot fungus *Gloeophyllum trabeum* were tested. Masson’s pine and poplar were selected as feed wood and control wood, respectively. Both measured 20 mm × 20 mm × 10 mm (T × R × L). All samples, including control wood, were weighed, and their dry weights were recorded before they were placed in a flask inoculated with decay fungi. After 8 weeks of infection with decay fungi at a temperature of 25 ± 2 °C and a humidity of 85 ± 5%, the dry weights of all samples were measured again, and the weight-loss rate of each sample was obtained according to the following equation:

(7) $$\mathrm{WLR}=\frac{M_{1}-M_{2}}{M_{1}}\times 100\%$$

where WLR is the mass-loss ratio (%), and *M*_1_ (g) and *M*_2_ (g) are the weights of the samples before and after infection, respectively.

### 2.6. Thermal Stability

The thermal stability of untreated and furfurylated BSCs was evaluated using thermal analyzer (NETZSCH STA 449F3, NETZSCH Gertebau GmbH, Selb, Germany) to determine TG and DTG values.

### 2.7. Characterization of Changes in Chemical Composition

Fourier-transform infrared spectroscopy (Nicolet-iS10, Thermo Scientific, Waltham, MA, USA) and XPS (Escalab 250Xi, Thermo Fisher Scientific, Waltham, MA, USA) were employed to analyze the functional groups and the O/C ratios, respectively, of untreated BSC, and furfurylated BSCs.

## 3. Results and Discussion

### 3.1. Physical Properties

**Figure 1:**
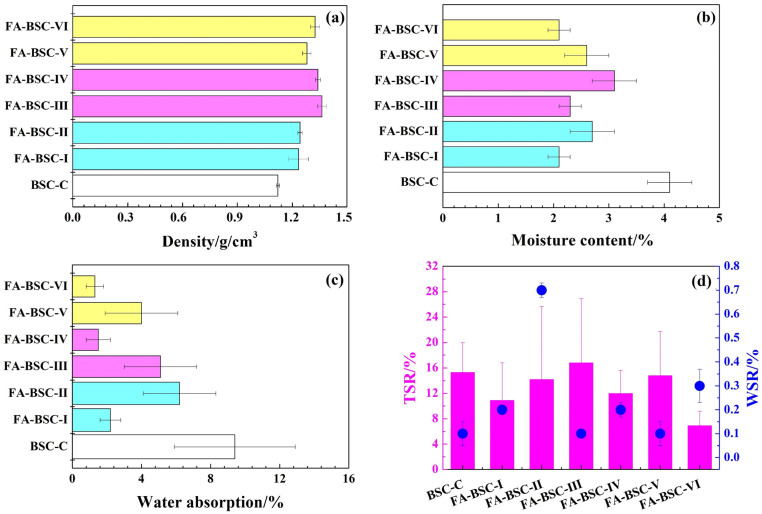
Physical properties of untreated BSC and furfurylated BSC. (**a**) density; (**b**) moisture content; (**c**) water absorption; (**d**) thickness and width swelling rate.

The physical properties of the untreated BSC and the BSCs furfurylated by different processes were measured (Figure 1). In this study, the method of preparing the same density was not selected, but the same paving method and the same number of layers were selected to prepare the BSCs. As shown in Figure 1a, the density of the BSC-C was 1.121 g/cm^3^, which was lower than that of the furfurylated BSCs. The highest density of the BSC that was furfurylated with 50% FA and cured at 105 °C through V–P–V impregnation was 1.362 g/cm^3^. In general, the density of the BSC modified with 50% FA was higher than that of the BSC modified with 30% FA. Furthermore, the density of the BSC impregnated by the V–P–V process was higher than that of the BSC impregnated by the soaking process, even though the soaking time (24 h/48 h) was significantly longer than the pressure-treatment time (10 min). The higher the concentration of the FA in the modified solution, the higher the weight-gain rate of the modified BSC. The efficiency of vacuum-pressure impregnation is higher than that of soaking treatment.

Bamboo is a naturally porous material containing many hydroxyl groups that absorb moisture and water through hydrogen bonds [31]. Previous studies found that furfurylation can significantly reduce the hygroscopicity of bamboo [24,26]. Figure 1b shows that the average moisture content of the BSC-C was approximately 4%, while that of the furfurylated BSCs was only 2–3%. Similarly, the water absorption of the furfurylated BSCs was also significantly lower than that of the untreated BSC, as shown in Figure 1c. This was mainly because the BSC was a relatively high-density plate made by hot-pressing the bamboo bundles. After the furfurylation, the hydrophobic FA resin filled the bamboo-cell cavities and even the micro-pores of the cell wall, which further prevented the water molecules from reaching the hydrophilic polymers in the bamboo-cell walls. The dimensional stability of the BSC samples was characterized by TSR and WSR. The TSR and WSR of the untreated BSC were 15.3% and 0.1%, respectively; the corresponding values for most of the furfurylated BSCs decreased, except for the TSR of the FA-BSC-III (16.8%) and the WSR of the FA-BSC-II (0.7%), which were slightly higher. For the FA-BSC-III sample, which had the highest weight-gain rate, the TSR was higher. This result may have been due to the cracking of the inter-layer adhesive layer during the boiling (4 h), roasting (20 h), and boiling (4 h) processes of the furfurylated BSCs, leading to an increase in thickness expansion.

### 3.2. Mechanical Properties

**Figure 2:**
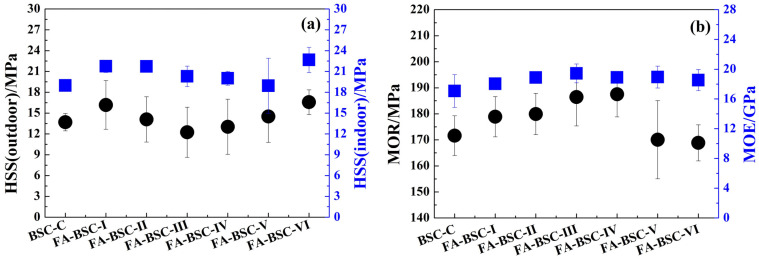
Mechanical properties of untreated BSC and furfurylated BSC. (**a**) HSS for outdoor and indoors; (**b**) MOR and MOE.

The HSS of the BSCs for both outdoor and indoor use were measured (Figure 2a). The HSSs of the untreated BSC were 13.7 MPa and 19.0 MPa for outdoor and indoor use, respectively. In contrast, the HSS of the furfurylated BSCs for indoor use increased slightly compared with that of the untreated bamboo. In particular, the HSS of the FA-BSC-VI, that is, of the bamboo bundles soaked in 50% modified FA solution for 48 h, increased by 21% compared with that of the untreated BSC. For outdoor use, the HSSs of some of the furfurylated BSCs with higher weight-gain rates were lower than that of the untreated BSC. The FA-BSC-III samples had the lowest HSSs, approximately 10% lower than that of the untreated BSC. These results indicate that the bonding strength of the furfurylated BSCs was affected during the boiling (4 h), roasting (20 h), and boiling (4 h) processes.

The MOR and MOE of the BSCs are shown in Figure 2b. The average MOR and MOE values of the untreated BSC were 171.6 MPa and 17.1 GPa, respectively. The furfurylation had little effect on the MOE of the bamboo scrimber but had an obvious effect on its MOR. The MOR of the furfurylated BSCs in the different impregnation processes differed. The MOR of the furfurylated BSCs prepared from the bamboo bundles treated with V–P–V impregnation was higher than that of the untreated BSC. The MOR of the BSCs prepared by using the soaking treatment was slightly lower than that of the untreated BSC. This effect could be explained by the filling of the FA resin in bamboo cell cavities and the bulking of the FA resin in the bamboo-cell wall. Proper filling and bulking could improve the mechanical strength of the material, but excessive bulking would reduce the mechanical strength of the material. In our previous research, we found that the MOR of bamboo furfurylated with 15% FA was significantly higher than that of untreated bamboo, while that of bamboo furfurylated with 50% FA was lower than that of untreated bamboo [24]. In addition, in this study, the properties of the modified and untreated BSC with the same paving mode were compared, instead of the properties of materials with the same density. The density of the modified BSCs was significantly higher than that of the untreated BSC, which was also a reason for the increase in the mechanical strength compared with the untreated BSC.

### 3.3. Durability

#### 3.3.1. Resistance to Mold Fungi

The mold-resistance-test results of the untreated and furfurylated bamboo scrimbers are shown in Table 4. The mold-prevention efficacy of the BSC-C was zero for the four types of mold fungi selected, which illustrated that it was highly susceptible to mold. In contrast, the mold resistance of the furfurylated BSCs significantly improved. The mold-prevention efficacy of the furfurylated BSCs was more than 75% against *A. niger* and *T. viride*. The prevention efficacy of the FA-BSC-Ⅲ, FA-BSC-Ⅴ, and FA-BSC-VI against *P. citrinum* also improved to some extent. The mold resistance of the furfurylated BSCs with 50% FA was slightly better than that of the furfurylated BSCs with 30% FA. Furfurylation is not sufficient to improve resistance to *B. theobromae*, one of the major fungi causing the discoloration of wood and bamboo, and this requires further investigation in follow-up studies.

#### 3.3.2. Resistance to Decay Fungi

Bamboo scrimber is believed to possess excellent decay resistance, especially against basidiomycetes [32]. *Trametes versicolor* (white rot) and *Serpula lacrymans* (brown rot) were used to test the decay resistance of bamboo scrimber, and the results showed that bamboo scrimber can be classified as highly resistant to both fungi [33]. Table 5 lists the weight-loss ratio of the untreated and furfurylated BSCs after 8 weeks of erosion by white-rot (*Coriolus versicolor*) and brown-rot (*Gloeophyllum trabeum*) fungi. The weight-loss ratio of poplar, as the control sample in this test, was 72.8% and 63.2% after infection with *C. versicolor* and *G. trabeum*, respectively, which illustrated that the anti-decay test was effective. The decay resistance of the BSCs was further improved by the furfurylation. The weight-loss ratio of the BSC-C was 12.2%, belonging to class II (decay resistance), while the furfurylated BSCs exhibited weight-loss ratios lower than 10% and were categorized as class I (strong decay resistance). This was related to the change in the BSC moisture and structure. The microstructure analysis found that the porosity of the BSC was significantly reduced by the furfurylation. This not only prevented the flow of water molecules in and out of the BSC, but also effectively prevented the appearance of fungal hyphae in the BSCs.

### 3.4. Microstructure

**Figure 3:**
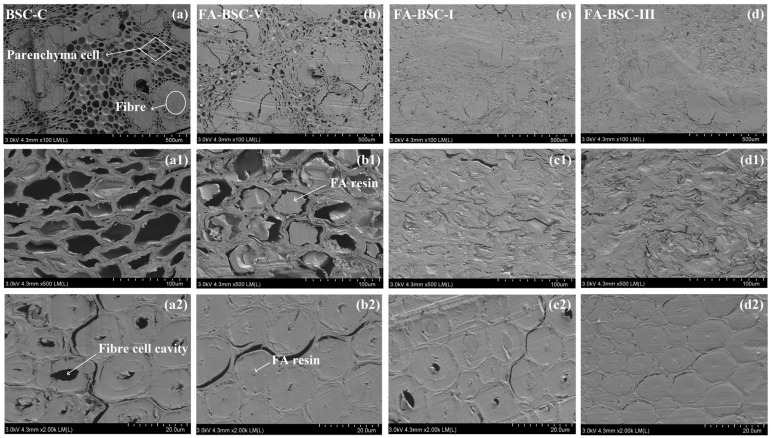
SEM images of untreated and furfurylated BSC cross-sections: (**a**,**a1**,**a2**) from BSC-C; (**b**,**b1**,**b2**) from FA-BSC-V; (**c**,**c1**,**c2**) from FA-BSC-I; (**d**,**d1**,**d2**) from FA-BSC-III. (**a1**,**b1**,**c1**,**d1**) Parenchyma cells. (**a2**,**b2**,**c2**,**d2**) Fiber cells.

Figure 3 compares the microstructural differences between the cross-sections of the untreated and furfurylated BSCs. Bamboo mainly consists of vascular bundles (fiber cells and vessel cells) surrounded by basic tissues (parenchyma cells) [34]. The SEM analysis revealed that almost all the cells were compressed and deformed to varying degrees. The deformation of the parenchyma cells and vessel cells was particularly obvious. As shown in Figure 3a1,a2, the parenchyma cells of the untreated BSC were empty and compressed from their original round shape to an oval shape, and some even closed. Although the deformation of the fiber cells was relatively small, some cells also showed cell-wall collapse or interlayer tearing. This was consistent with previous studies. Rao et al. [10] and Li et al. [35] reported that the thin-walled vessel cells and parenchyma cells of bamboo were compressed or even closed, and that the thick-walled fiber cells remained essentially unchanged. Compared with the untreated BSC, the parenchyma cells of the furfurylated BSCs were filled with large quantities of FA resin, and the parenchyma cells were compressed and deformed more significantly, as shown in Figure 3b1,c1,d1. This indicates that the dispersed bamboo bundles, which were the bamboo-based units used to prepare the BSCs, were easily permeated by the furfuryl alcohol. In addition, furfurylation may cause thin-walled cells, such as parenchyma cells, to become compressed more easily. Moreover, some of the fiber cells were also filled, but they remained almost intact, as shown in Figure 3b2,c2,d2. This indicates that a certain amount of FA-resin filling plays a particular role in supporting thick-walled cells, such as fiber cells. It was further observed that the furfurylation reduced the porosity and increased the density of the BSCs, which may have been the reason for the enhanced mechanical properties and durability and decreased water absorption of the BSCs. Interestingly, when the compression state of the parenchyma-cell walls (Figure 3c2,d2) were observed, the bamboo-parenchyma-cell walls showed ductile deformation during the compression process, and the bamboo-cell walls did not become brittle or collapse due to the furfurylation. This indicated that the degradation of the components of the bamboo-cell walls was not very serious after the furfurylation, and the parenchyma-cell walls maintained good flexibility.

### 3.5. Chemical Analysis

#### 3.5.1. FTIR

**Figure 4:**
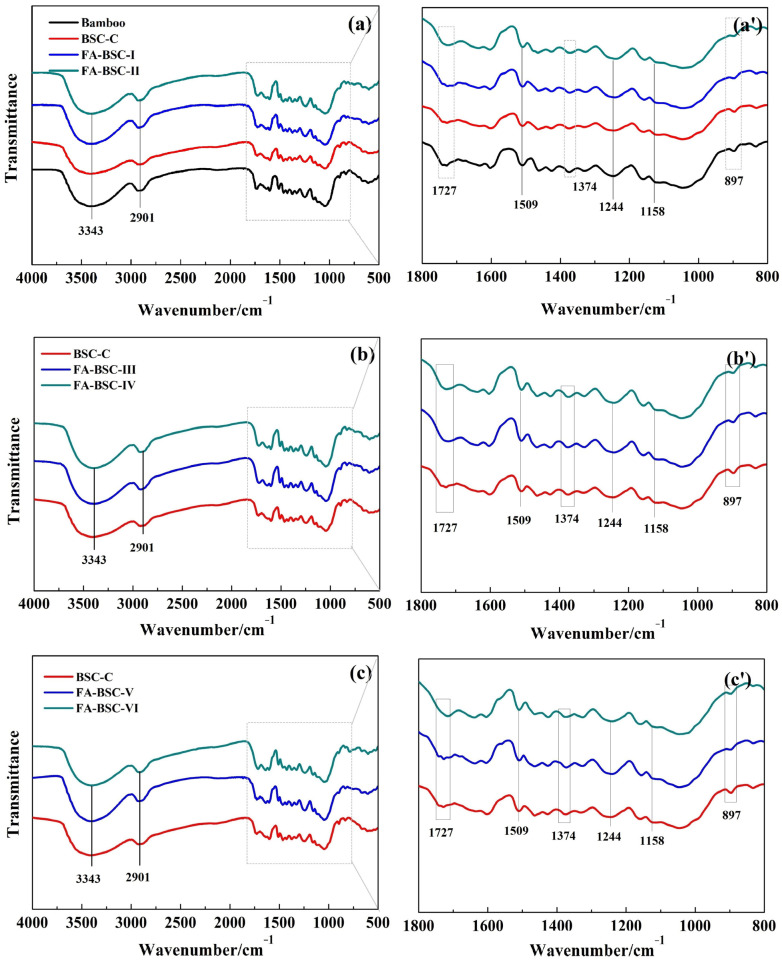
FTIR spectra of bamboo, untreated BSCs, and furfurylated BSCs. Peak assignment: 3343 cm^−1^ assigned to O-H stretching vibrations (cellulose), 2901 cm^−1^ assigned to alkyl group (-CH_2_), 1727 cm^−1^ attributed to the stretching of acetyl and carboxyl acid (hemicellulose), 1509 cm^−1^ assigned to the aromatic skeletal vibrations in lignin, 1374 cm^−1^ assigned to the C-H deformation bonds (cellulose and hemicellulose), 1244 cm^−1^ assigned to the C-O stretching of the guaiacyl ring in lignin, 1158 cm^−1^ assigned to the C-O-C bond, 897 cm^−1^ assigned to the structural contribution of cellulose and hemicellulose. (**a′**,**b′**,**c′**) was the fingerprint region in the range of 800–1800 cm^−1^ of (**a**,**b**,**c**), respectively.

To study the chemical-functional-group changes to the bamboo, bamboo scrimber, and furfurylated bamboo scrimber, FTIR spectra of the three materials were collected, as shown in Figure 4. The intensities of the absorption peaks at 3343 cm^−1^ assigned to the O-H stretching vibrations (cellulose) decreased in the bamboo scrimber, and further decreased in the furfurylated BSCs. This result was due to the dehydration polycondensation of the free hydroxyl group between the molecular chains of the bamboo cellulose during the hot-pressing process. Furthermore, some of the hydroxyl groups may have etherified with the modified furfuryl-alcohol solution, resulting in the further reduction of the hydroxyl groups. This may explain why the furfurylation significantly reduced the moisture content and water absorption of the bamboo scrimber. In general, slight changes in the fingerprint region in the range of 800–1800 cm^−1^ were observed. To more clearly compare the differences between the fingerprint-region spectra of the three materials, the calibration-fit method, proposed by Rodrigues et al. [36], was applied. The ratio of the average relative intensities of the lignin peak height at 1509 and 1244 cm^−1^ to those of the carbohydrate peak height at 1727, 1374, 1158, and 897 cm^−1^ is presented in Table 6. The peaks at 1509 and 1244 cm^−1^ were assigned to the aromatic skeletal vibrations and the C-O stretching of the guaiacyl ring in the lignin. The peaks observed at 1727 cm^−1^, 1374 cm^−1^, 1158 cm^−1^, and 897 cm^−1^ corresponded to the stretching of the acetyl and carboxyl acid (hemicellulose), the C-H deformation bonds (cellulose and hemicellulose), the C-O-C bond, and the structural contribution of the cellulose and hemicellulose [35,37]. The values of I_1158_/I_1509_ and I_1244_/I_1727_ in the bamboo were 0.64 and 0.73, respectively, and increased to 0.92 and 0.9 in the bamboo scrimber, respectively, but the corresponding values of the furfurylated BSCs increased slightly. The peaks at 1158 and 1244 cm^−1^ were attributed to the cellulose and the stretching in the phenol–ether bond, respectively. This increase was probably due to the hydroxyl group of the carboxylic acid binding to the hydroxyl group of the PF and the FA resin. The ratio of I_897_/I_1509_ in the bamboo was 1.36; it decreased to 1.18 in the bamboo scrimber, and further decreased to about 1.07 after the furfurylation. The peak at 897 cm^−1^ was assigned to the structural contribution of the cellulose and hemicellulose. This reduction indicates that the cellulose and hemicellulose in bamboo may be hydrolyzed to some extent during the process of bamboo-scrimber preparation and furfurylation.

#### 3.5.2. XPS

**Figure 5:**
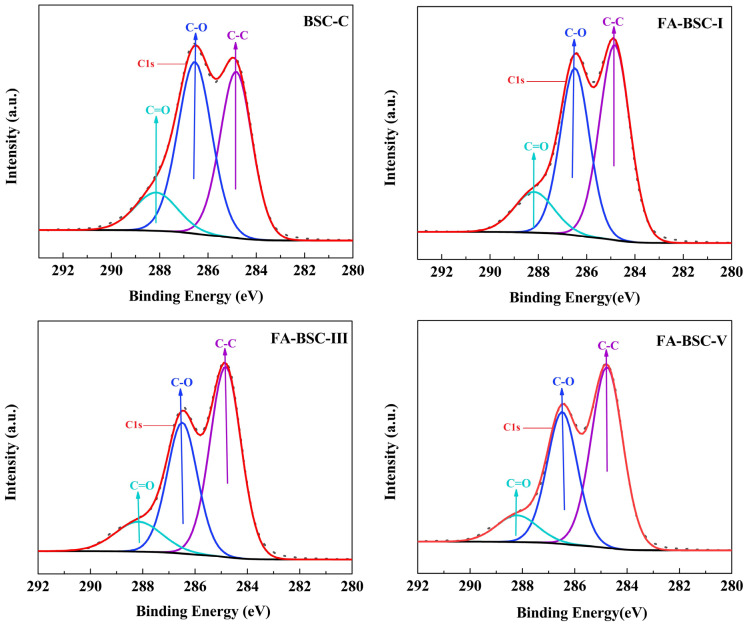
XPS spectra of C peaks in untreated and furfurylated BSCs.

**Figure 6:**
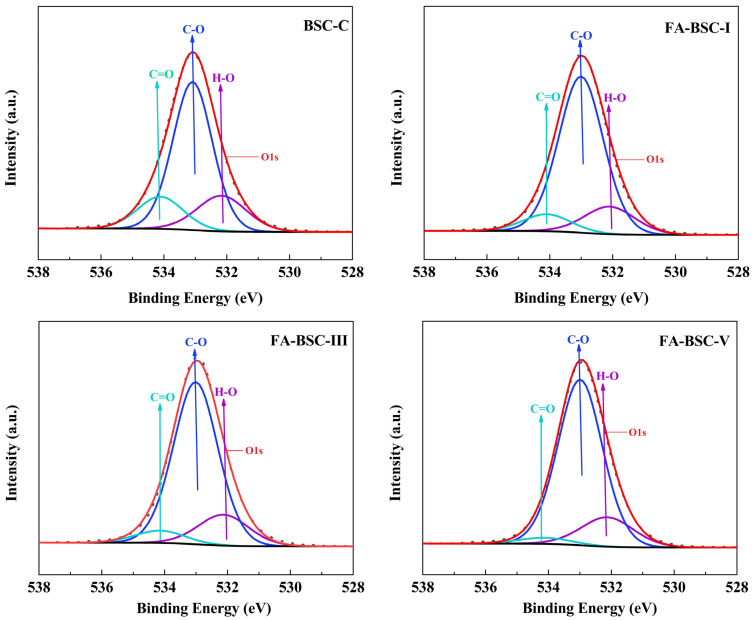
XPS spectra of O peaks in untreated and furfurylated BSCs.

The effect of the furfurylation on the chemical properties of the BSCs was further evaluated by XPS. As shown in Figure 5 and Figure 6, both the C1s and the O1s XPS spectra of the samples were deconvoluted into three components. The former were C_1_, C_2_, and C_3_, associated with C-C, C-O, and C=O, respectively [38,39,40]. The latter were O_1_, O_2_, and O_3_, associated with H-O, C-O, and C=O, respectively [35,40,41]. The oxygen-to-carbon (O/C) ratio and the relative composition distribution of the oxygen and carbon atoms are presented in Table 7. It is clear that the C_1_ and O_2_ components of the BSCs increased after the furfurylation, while the C_2_, O_1_, and O_3_ components decreased because the FA polymerization introduced a large number of C-C and C-H bonds into the bamboo structure. Furthermore, the increase in O_2_ was probably related to the dehydration condensation reaction between the hydroxyl group of carboxylic acid and the hydroxyl group of the furan ring [42]. In contrast, the C_2_ and O_3_ components of the furfurylated BSCs from the cellulose and hemicellulose were significantly reduced, which may have been due to the acid hydrolysis of the cellulose and hemicellulose during the furfurylation. There were moderate decreases in C_3_ and O_1_. These changes were mainly caused by the decrease in the hemicellulose and cellulose contents and the increase in the lignin content after the furfurylation of the BSCs. The O/C ratio of the BSC decreased after the furfurylation. Similar results were reported for furfurylated and heat-treated bamboo [37,43]. Matuana et al. [44] suggested that C_1_ represents unoxygenated carbons, while C_2_-C_4_ represents oxygenated carbons, and that the C_ox_/C_unox_ ratio can be calculated using the following formula: (C_2_ + C_3_ + C_4_)/C_1_. Table 7 shows that the furfurylation reduced the Cox/Cunox ratio of the BSCs, which was attributed to the introduction of more unoxygenated carbon by the FA polymers. In general, the XPS results were consistent with the changes observed through the FTIR spectra.

### 3.6. Analysis of Thermal-Degradation Behavior 

**Figure 7:**
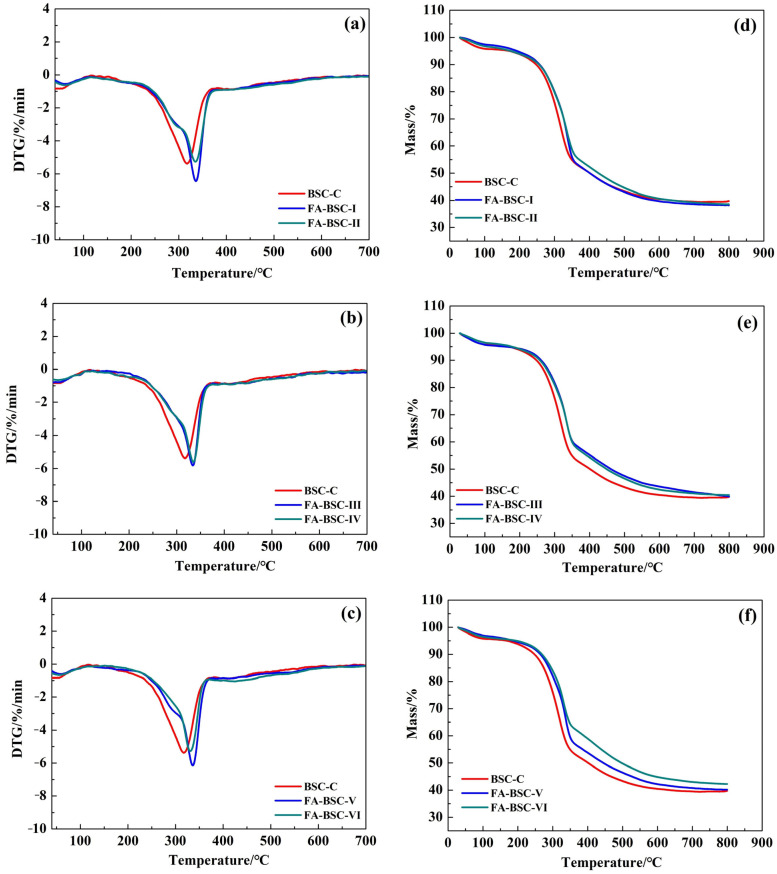
Thermogravimetric analysis of untreated BSC and furfurylated BSCs. (**a**,**d**) from BSC-C, FA-BSC-I and FA-BSC-II; (**b**,**e**) from BSC-C, FA-BSC-III and FA-BSC-IV; (**c**,**f**) from BSC-C, FA-BSC-V and FA-BSC-VI.

Figure 7 shows the results of the thermogravimetric analysis of the untreated and furfurylated BSCs. The thermogravimetric profiles mainly consisted of three stages. The first stage was between 30 and 160 °C; it featured minor weight loss, which was mainly associated with the removal of free water and bound water. The second stage refers to the decomposition of the hemicellulose and unstable cellulose. The weight loss mainly occurred in this stage, in the temperature range of 160–340 °C, with a corresponding weight loss of 38.32% in the BSC-C and a weight-loss range of 28.50 to 35.94% in the furfurylated BSCs. The last stage consisted of a slowdown in the thermal decomposition between 340 and 800 °C. The remaining residue at 800 °C for the BSC-C was 39.6%, and for the furfurylated BSCs, it ranged from 38.1 to 42.2%. Similar results were reported for the furfurylated bamboo samples. Liu et al. (2022) [37] reported that the weight residue of furfurylated bamboo was 18%, while the weight residue of their control was 13%. These results indicated that the furfurylation increased the weight residue and improved the thermal stability of the BSCs to a certain extent by filling the materials with FA resin. Additionally, the maximum DTG cure was attained at approximately 320 °C for the untreated BSCs and 335 °C for the furfurylated BSCs, which further indicated that the furfurylation increased the thermal stability of the BSCs. For the BSCs furfurylated by different processes, the peak temperatures of the DTG curves were almost equal, but the maximum weight-loss rate and residual mass were slightly different. When the temperature was 335 °C, the weight-loss rates of the furfurylated BSCs were ranked as follows, from high to low: FA-BSC-I > FA-BSC-II > FA-BSC-III = FA-BSC-IV > FA-BSC-V > FA-BSC-VI. This was attributed to the incorporation of the FA polymer into the bamboo structure.

## 4. Conclusions

This study evaluated the physical–mechanical properties and durability of furfurylated BSCs with different impregnation processes, different FA concentrations, and different curing temperatures. The furfurylated BSCs had a higher density, lower moisture content and water-absorption rate, and better dimensional stability than the untreated BSC. The improvements in these properties were directly related to the changes in the microstructure. As observed using SEM, the FA resin effectively filled in the bamboo-cell cavities and vessels, and the modified bamboo-parenchyma cells were compressed more tightly and evenly. In terms of the mechanical properties, the furfurylation had a slight negative effect on the mechanical strength of the BSCs, and the MOR and HSS of the furfurylated BSCs increased to a certain extent under most of the treatment conditions, although the FTIR and XPS spectroscopy showed that the cellulose and hemicellulose underwent acid hydrolysis to a certain extent after the furfurylation. This may have been related to the relatively high density of the furfurylated BSCs. In addition, the furfurylation significantly improved the mold and decay resistance of the BSCs, especially the decay resistance, which was raised from decay resistance (class II) to strong decay resistance (class I). The present study highlights the potential of furfurylation as a modification method to enhance BSC products. Based on the results of this study, a combination of the V–P–V impregnation process, a concentration of FA of 50%, and a curing temperature of 115 °C was proposed as the set of starting processing parameters for the furfurylation of bamboo-scrimber composites. Further research should focus on improving the ability of furfurylated BSCs to prevent staining fungi. Additionally, the influence of FA resin on the bonding strength of PF adhesives should be further clarified.

## Figures and Tables

**Table 1:** Abbreviation and processing-technology information of samples.

Abbreviation	Samples	Treatment of Bamboo Bundles	Impregnation Times	Curing Temperature
Bamboo	Bamboo bundles	untreated		
BSC-C	Bamboo-scrimber composites	untreated		
FA-BSC-Ⅰ		furfurylated with 30% FA through V–P–V impregnation	10 min vacuum +10 min pressure +10 min vacuum	105 ℃
FA-BSC-Ⅱ				115 ℃
FA-BSC-Ⅲ		furfurylated with 50% FA through V–P–V impregnation		105 ℃
FA-BSC-Ⅳ				115 ℃
FA-BSC-Ⅴ		furfurylated with 50% FA through soaking impregnation	24 h	105 ℃
FA-BSC-Ⅵ			48 h	105 ℃

**Table 2:** Dimensions and numbers of BSC specimens used for physical–mechanical and durability measurements.

Items	Size (L × T × R)	Numbers for Each Group
TSR and WSR	50 mm × 50 mm × 18 mm	8
HSS	108 mm × 40 mm × 18 mm	6
MOR and MOE	330 mm× 20 mm× 18 mm	5
Resistance to mold	50 mm × 20 mm × 10 mm	24
Resistance to decay	20 mm × 20 mm × 10 mm	24
Resistance to termites	25 mm × 25 mm × 6 mm	5

**Table 3:** Reference for evaluation of infection value.

Infection Value	Sample Infected Area
0	No visible growth
1	Mold infection covered less than 1/4 of the surface of the sample
2	Mold infection covered between 1/4 and 1/2 of the surface of the sample
3	Mold infection covered between 1/2 and 3/4 of the surface of the sample
4	Mold infection covered more than 3/4 of the surface of the sample

**Table 4:** Mold-prevention efficacy of BSCs.

Samples	Mold-Prevention Efficacy (%)			
	*Aspergillus niger* V. Tiegh	*Penicillium citrinum* Thom	*Trichoderma viride* Pers. ex Fr	*Botryodiplodia theobromae* Pat
BSC-C	0	0	0	0
FA-BSC-Ⅰ	75	25	100	0
FA-BSC-Ⅱ	75	0	75	0
FA-BSC-Ⅲ	75	75	75	25
FA-BSC-Ⅳ	75	75	75	0
FA-BSC-Ⅴ	75	75	100	25
FA-BSC-Ⅵ	100	50	100	75

**Table 5:** Weight-loss ratio of untreated and furfurylated bamboo scrimber after decay-resistance test.

Samples	Weight-Loss Ratio (%)		Resistance Level
	*Coriolus versicolor*	*Gloeophyllum trabeum*	
BSC-C	12.2	12.2	Ⅱ
FA-BSC-Ⅰ	7.62	7.04	Ⅰ
FA-BSC-Ⅱ	6.81	6.49	
FA-BSC-Ⅲ	8.35	7.41	
FA-BSC-Ⅳ	6.70	7.50	
FA-BSC-Ⅴ	6.11	5.72	
FA-BSC-Ⅵ	5.51	5.42	
Poplar	72.8	63.2	Ⅳ

Note: weight-loss ratio < 10%, strong decay resistance (Ⅰ); 11% < weight-loss ratio < 24%, decay resistance (Ⅱ); 25% < weight-loss ratio < 44%, slight decay resistance (Ⅲ); weight-loss ratio > 45%, no resistance (Ⅳ).

**Table 6:** Ratios of the intensities of lignin-associated bands to those of carbohydrate bands for BSCs.

	Bamboo	BSC-C	FA-BSC-I	FA-BSC-II	FA-BSC-III	FA-BSC-IV	FA-BSC-V	FA-BSC-VI
I_1727_/I_1509_	0.99	1.04	1.00	1.01	1.00	1.00	1.01	0.98
I_1374_/I_1509_	0.82	0.97	0.95	0.96	0.96	0.96	0.95	0.96
I_1158_/I_1509_	0.64	0.92	0.89	0.92	0.90	0.92	0.90	0.93
I_897_/I_1509_	1.36	1.18	1.07	1.07	1.12	1.08	1.07	1.08
I_1509_/I_1727_	1.01	0.96	1.00	0.99	1.00	1.00	0.99	1.02
I_1244_/I_1727_	0.73	0.90	0.90	0.92	0.92	0.93	0.90	0.96

**Table 7:** Relative atomic compositions of BSCs.

Element Component	Binding Energy (eV)	Bond	Main Resource	BSC-C	FA-BSC-I	FA-BSC-Ⅲ	FA-BSC-V
O/C				0.45	0.44	0.41	0.41
C_1_	284.84	C-C	Lignin, fatty acids, and other extractives	41.61	47.67	52.23	52.69
C_2_	286.55	C-O	Cellulose and hemicellulose	45.59	39.25	35.23	37.11
C_3_	288.14	C=O	Hemicellulose	12.8	13.08	12.54	10.2
C_ox_/C_unox_ ratio				1.4	1.1	0.91	0.9
O_1_	532.17	H-O	Lignin	19.31	15.08	16.54	16.61
O_2_	533.09	C-O	Cellulose and hemicellulose	64.49	76	76.91	79.96
O_3_	534.11	C=O	Lignin	16.2	8.92	6.55	3.43

## Data Availability

The data presented in this study are available on request from the corresponding author.
